# Evaluation of Short-Term Postoperative Outcomes of Lateral Lymph Node Dissection After Neoadjuvant Radiotherapy for Rectal Cancer Patients: The Early Learning Phase After Surgical Training in the Netherlands

**DOI:** 10.1245/s10434-025-17155-6

**Published:** 2025-05-08

**Authors:** Eline G. M. van Geffen, Tsuyoshi Konishi, Sanne-Marije J. A. Hazen, Tania C. Sluckin, Charmaine M. Tjin-A-Koeng, Eric H. J. Belgers, Johanna G. Bloemen, Esther C. J. Consten, Rogier M. P. H. Crolla, Michalda S. Dunker, Klaas Havenga, Christiaan Hoff, Fatih Polat, Maria Verseveld, Karin Horsthuis, Pieter J. Tanis, Miranda Kusters

**Affiliations:** 1 Department of Surgery, Amsterdam UMC location Vrije Universiteit Amsterdam, Amsterdam, The Netherlands; 2 Treatment and Quality of Life, Imaging and Biomarkers, Cancer Center Amsterdam, Amsterdam, The Netherlands; 3 Department of Colon and Rectal Surgery, The University of Texas MD Anderson Cancer Center, Houston, TX USA; 4 Department of Surgery, Zuyderland Medical Centre, Heerlen, The Netherlands; 5 Department of Surgery, Catharina Hospital Eindhoven, Eindhoven, The Netherlands; 6 Department of Surgery, Meander Medical Centre, Amersfoort, The Netherlands; 7 Department of Surgery, Amphia Hospital, Breda, The Netherlands; 8 Department of Surgery, Northwest Clinics, Alkmaar, The Netherlands; 9 Department of Surgery, University Medical Center Groningen, University of Groningen, Groningen, The Netherlands; 10 Department of Surgery, Medical Centre Leeuwarden, Leeuwarden, The Netherlands; 11 Department of Surgery, CWZ Nijmegen, Nijmegen, The Netherlands; 12 Department of Surgery, Franciscus Gasthuis and Vlietland, Rotterdam, The Netherlands; 13 Department of Radiology and Nuclear Medicine, Amsterdam UMC location Vrije Universiteit Amsterdam, Amsterdam, The Netherlands; 14 Department of Surgical Oncology and Gastrointestinal Surgery, Erasmus MC, Rotterdam, The Netherlands; 15 Department of Surgery, Amsterdam UMC location AMC, Amsterdam, The Netherlands

## Abstract

**Background:**

Distal, locally advanced rectal cancer might spread to lateral lymph nodes (LLNs), posing a risk of lateral local recurrence (LLR). This study evaluated quality-controlled implementation of lateral lymph node dissection (LLND) in the Netherlands.

**Methods:**

This retrospective multicenter cohort study included consecutively treated rectal cancer patients who underwent neoadjuvant therapy, total mesorectal excision (TME) surgery, and nerve-sparing minimally invasive LLND by trained surgeons across 10 Dutch hospitals. Training involved cadaver sessions, monthly video meetings, and proctoring. Outcome measures included intra- and postoperative complications, urogenital dysfunction and 18-month LLR, local recurrence (LR), and disease-free survival (DFS).

**Results:**

The study comprised 41 patients (median follow-up period, 16 months; interquartile range, IQR, 8–21 months) with advanced tumors (27% cT4, 49% cN2, 7% cM1), and a mean LLN size of 11 mm on primary-staging MRI. Abdominoperineal resection was performed for 29 patients (70%). A beyond TME procedure was performed for 11 patients (28%). The median blood-loss was 250 ml (IQR, 100–400 ml), with obturator nerve injury reported in one patient. Malignant LLNs were found in 41% of the LLND specimens. Complications occurred for 22 patients (54%), 21% (9/41) of which were grade 3 or higher. Nine patients (22%, four of whom underwent beyond TME surgery) had a Foley or intermittent urinary catheter at the end of the follow-up period. Sexual dysfunction of three patients was reported. No ipsilateral LLRs occurred. The 18-month LR rate was 14%, and the DFS was 55%.

**Conclusion:**

Minimally invasive nerve-sparing LLND by trained Dutch surgeons showed acceptable complication rates and good oncologic control of the lateral compartment to date.

In approximately 4% of rectal cancer patients treated with curative intent, cancer spreads to the lateral lymph nodes (LLNs) located around the internal iliac and obturator vessels.^1,2^ As shown on primary MRI, these LLNs become enlarged (≥7 mm), which is associated with a high 5-year risk of lateral local recurrence (LLR) reaching 20% despite neoadjuvant (chemo)radiation ([C]RT) and total mesorectal excision (TME).^3,4^ Lateral lymph node dissection (LLND) as an addition to ([C]RT and TME is thought to lower this risk.^5,6^

In Japan and other Eastern countries, this procedure has been widely adopted, and minimally invasive, nerve-sparing unilateral LLND seems not to worsen functional outcomes.^7–9^ Nevertheless, Dutch surgeons remain apprehensive about the procedure due to concerns about possible urogenital dysfunction, obturator nerve damage, and blood loss^5,10–12^ associated with the procedure, especially in a Western population wherein obesity is more common,^13^ and prior radiotherapy is standard practice for patients with extra-mesorectal lymph nodes.^14–18^

This topic was discussed during several meetings endorsed by the Dutch Society of Colorectal Surgery. It was concluded that to gain oncologic benefit from the procedure while minimizing the post-procedural complications, LLND surgery must be centralized. A few colorectal surgeons from each region in the Netherlands were subsequently trained in a minimally invasive nerve-sparing technique.^19^ Moreover, radiologists have been trained in recognition, measurement, and classification of LLNs,^20^ and radiation oncologists have been trained in tailored radiation of the lateral compartments according to updated national guidelines.^21^ This multidisciplinary collaboration after dedicated training of radiation oncologists, radiologists, and pathologists will be evaluated in the currently recruiting prospective LaNoReC study.^19^

The current study aimed to determine surgical and mid-term oncologic outcomes from the quality-controlled implementation of minimally invasive nerve-sparing LLND in the Netherlands.

## Methods

Data collection for this multicenter retrospective cohort study was conducted between April 2024 and June of 2024 in the Netherlands. All consecutive patients with neoadjuvant radiotherapy (with or without chemotherapy) who underwent LLND of the internal iliac and obturator compartment for primary rectal cancer after the surgical training (September to November 2021) and before the LaNoReC trial were included from 10 centers (“Appendix 1”). Selection for LLND was predominantly based on short-axis size of ≥7 mm on primary MRI. If the size remained ≥7 mm on restaging MRI, this was routinely considered an indication for LLND, and in case of downsizing with a short-axis size of <7 mm, LLND was performed based on shared decision-making, thereby weighing potential oncologic benefit versus additional mortality.

Eligible patients were identified by trained surgeons. Training of radiologists was not part of the study, nor was centralized review of MRI. Data, including radiologic, surgical, and oncologic variables, were retrospectively collected by electronic patient file assessment. The Medical Ethics Committee of the Amsterdam UMC approved this study and determined it to be exempt from the Dutch Medical Research Involving Human Subjects Act.

### LLND Training

After several consensus meetings organized by the Dutch Society of Colorectal Surgery, LLND surgery was centralized. This centralization aimed to ensure adequate surgical exposure for each trained surgeon given the low incidence of enlarged LLNs. After regional oncology group meetings, 16 surgeons were appointed by each region to attend two training days in September and November 2021. During these sessions, an expert in LLND surgery (Dr. Konishi) provided extensive and video-assisted lectures on the anatomy of the lateral compartments, the oncologic impact of LLNs, and examples of LLND surgery.

**Fig. 1:**
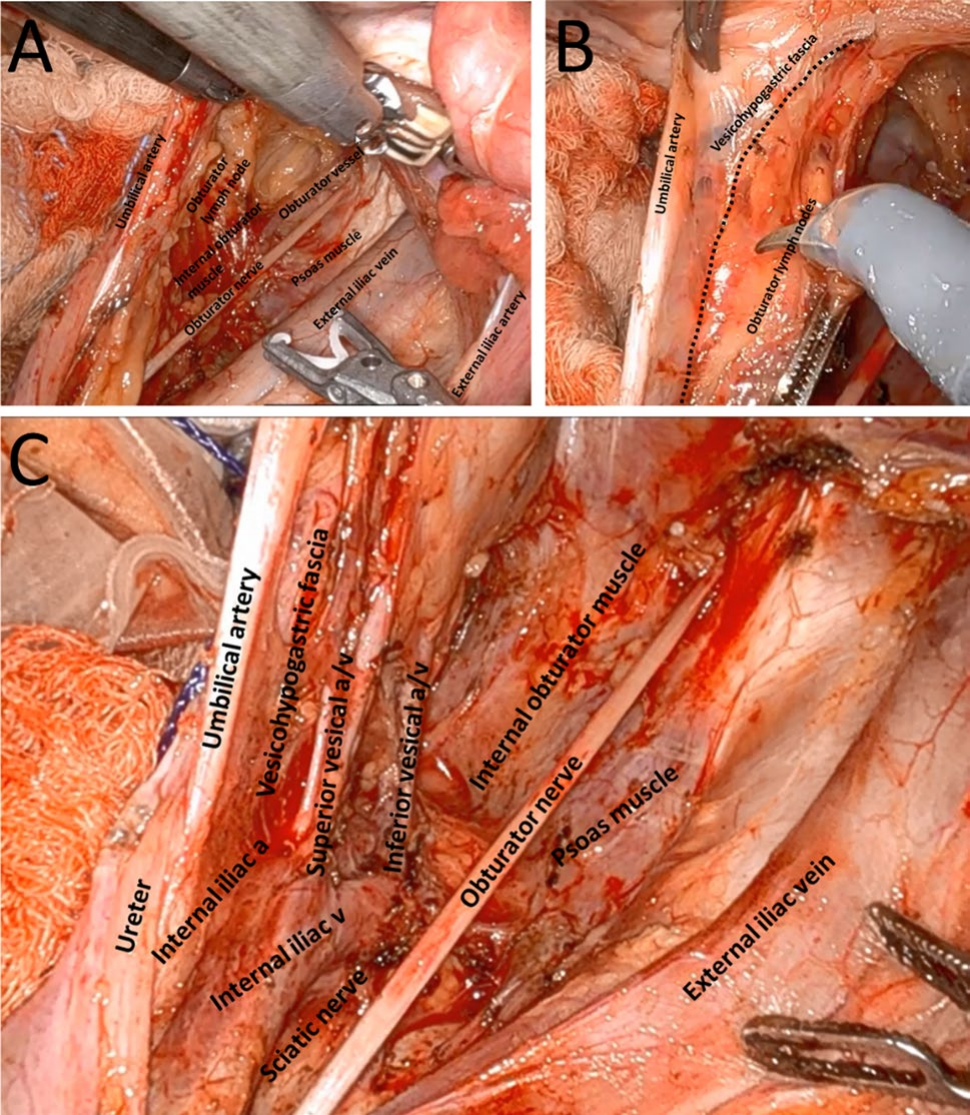
Intraoperative images of robot-assisted lateral lymph node dissection. **A** Overview of the lateral compartment during dissection of obturator lymph nodes. **B** The medial plane of lateral lymph node dissection as indicated by the umbilical artery and the vesicohypogastric fascia. **C** Overview of the lateral compartment after dissection of the internal iliac and obturator lymph nodes.

The surgeons practiced the minimally invasive, nerve-sparing technique in hands-on cadaver sessions, as previously outlined and illustrated in Fig. 1.^22,23^ During these sessions, the surgeons received live feedback from Dr. Konishi and their peers. Of the 16 surveons, 2 were already experienced in LLND surgery (1 in laparoscopic and 1 in robotic surgery), and were selected as proctors for further implementation. The remaining surgeons were paired geographically to ensure that each LLND procedure involved two trained surgeons. Many surgeons preferred local proctoring for their first few cases.

After these training sessions, monthly online meetings were held, with recorded LLND procedures reviewed and feedback provided by Dr. Konishi and the group. The meetings were recorded for a repeat review by surgeons and served also for presentation of new cases and discussion of preoperative planning.

### Outcome Measures and Definitions

The primary outcomes were a postoperative complication rate in fewer than 30 days and the presence of urinary dysfunction at the end of the follow-up period. For nerve damage of the obturator and hypogastric nerve, intraoperative reported damage and functional loss during the follow-up period were collected. Urinary dysfunction was assessed by the need for a urinary catheter at the end of the follow-up period or by functional urinary complaints reported in the electronic patient file. No standardized urinary function assessment was performed as part of this study, and data regarding urinary residual volume or flow were not collected. Sexual dysfunction at any point during the follow-up period was evaluated based on electronic patient files.

The postoperative complication rate encompassed any deviation from the expected recovery in fewer than 30 days. For each complication, the corresponding Clavien–Dindo classification (CDC) and date of occurrence were collected. The secondary outcomes included LLN characteristics observed on primary and restaging MRI, as well as pathologic findings such as lymph node harvest in the lateral compartment. The intraoperative variables included blood loss and damage to the hypogastric and obturator nerves. Surgical complications and duration of surgery also were assessed. Additionally, midterm postoperative outcomes were evaluated, including readmissions, 18-month rates of (L)LR, distant metastases (DM), disease-free survival (DFS), and overall survival (OS).

### Statistical Analysis

Categorical data are presented as frequencies and percentages. Numeric data are presented as mean with standard deviation (SD) or median with interquartile range (IQR), depending on the distribution. Kaplan-Meier analysis was used to describe (L)LR, DM, DFS, and OS rates. To compare subgroups, the chi-square or Mann–Witney *U* test was used, and significance was set at a *p* value lower than 0.05. All analyses were performed using IBM SPSS statistics version 28 (IBM, Chicago, IL, USA).

## Results

From November 2021 onward, 41 consecutive patients were enrolled in this study (“Appendix 1”). The median follow-up period was 16 months (IQR, 8–21 months). The number of resections performed by the surgeons in this cohort ranged from 2 to 20. Of these 41 procedures, 28 were either performed or proctored by a surgeon with prior experience. Among the remaining 13 procedures, 3 were performed in dual-surgeon teams, after which one of the two surgeons performed an additional 5 procedures. Two other surgeons performed LLNDs individually: the one performed two procedures, and the other performed three procedures.

Patient, tumor, and treatment characteristics are detailed in Table 1. Most of the patients were classified as ASA 2 (73%) and had a distally located tumor (median distance of 6 mm from the anorectal junction). The tumor stages were predominantly advanced (cT3, 66%; cT4, 27%), and most of the patients had suspected regional lymph nodes (cN1, 27%; cN2, 49%). Three patients (7%) had synchronous distant metastases at diagnosis, which were considered to be eligible for intentional curative treatment.

**Table 1:** Patient, tumor, and treatment characteristics

Whole cohort (*n* = 41)	*n* (%)		*n* (%)
Male sex	24 (59)	Initially watch and wait	9 (22)
Mean age at resection (years)	60 ± 12	Ostomy for obstruction preceding resection	5 (12)
Mean BMI (kg/m^2^)	27 ± 5	Type of surgery	
Previous abdominal surgery	10 (24)	TME with anastomosis	8 (20)
Previous malignancy	2 (5)	TME without anastomosis	2 (5)
ASA classification		APE	29 (70)
2	30 (73)	LLND alone (no TME)	2 (5)
3	11 (27)	Surgical approach	
Median distance from the ARJ: mm (IQR)^a^	6 (0–26)	Conventional laparoscopic	18 (44)
cT Stage		Robot-assisted laparoscopic	19 (46)
cT2	3 (7)	TaTME	4 (10)
cT3	27 (66)	Beyond TME	11 (28)
cT4	10 (27)	Partial prostate/vagina	4 (10)
Threatened MRF by primary tumor (≤1 mm)^b^	26 (65)	Seminal vesicle	4 (10)
cN Stage		Os coccyx/S5	1 (2)
cN0	10 (24)	Posterior exenteration	2 (5)
cN1	11 (27)	M. puborectalis	1 (2)
cN2	20 (49)	LLND surgery	
mrEMVI (grade 3 or 4)^c^	12 (32)	Unilateral	37 (90)
Lesion other than tumor ≤1 mm of MRF		Bilateral	4 (10)
No lesion/not reported	37 (90)	LLND resection	
mrEMVI	1 (2)	En bloc	6 (15)
mrTD	3 (7)	Separately	35 (85)
cM Stage		Total surgical time (min)^d^	330 ± 92
cM0/cMx	38 (93)	Median total blood loss: ml (IQR)^e^	250 (100–400)
cM1	3 (7)	Intraoperative complications	12 (29)
Neoadjuvant treatment		Bleeding (>500 ml)	4 (10)
Chemoradiation therapy	28 (68)	Ureter damage	2 (5)
Short course radiotherapy + consolidation therapy	8 (20)	Tumor perforation	2 (5)
Induction systemic therapy + chemoradiation therapy	4 (10)	Bladder perforation	1 (3)
Chemoradiation therapy + consolidation therapy	1 (2)	Vagina perforation	2 (5)
Radiotherapy boost	2 (5)	Damage to the obturator nerve	1 (3)

BMI, body mass index; ASA, American Society of Aaesthesiologists; ARJ, anorectal junction; IQR, interquartile range; MRF, mesorectal fascia; mrEMVI, MRI-detected extramural venous invasion; mrTD, MRI-detected tumor deposits; TME, total mesorectal excision; APE, abdominoperineal excision; LLND, lateral lymph node dissection; TaTME, transanal total mesorectal excision

^a^5 Missing values

^b^1 Missing value

^c^3 Missing values

^d^5 Missing values

^e^6 Missing values

### LLNs on Primary and Restaging MRI

The mean short-axis diameter of LLNs on primary MRI was 11 ± 5 mm; Table 2). In approximately half of the patients (*n* = 20, 49%), the largest LLN was located in the internal iliac compartment, whereas the remaining half (*n* = 21, 51%) had their largest LLN in the obturator compartment. Of the 41 patients, 11 patients (28%) had multiple enlarged LLNs on one side, and 3 (8%) had bilateral enlarged LLNs. Neoadjuvant therapy consisted mainly of CRT (68%; Table 1). Restaging MRI showed that the LLNs had decreased to a mean short-axis diameter of 8 ± 3 mm, with 22 (54%) remaining ≥7.0 mm in size and three measuring ≤4.0 mm.

**Table 2:** Lateral lymph node size on primary and restaging MRI

**Primary MRI**	**Whole cohort (*n* = 41**) ***n*** (%**)**
Median no. of LLNs (IQR)	1 (1–2)
Mean short-axis size of largest LLN (mm)	11 ± 5
LLN compartment of the largest LLN	
Internal iliac	20 (49)
Obturator	21 (51)
Short-axis size of largest LLN (mm)	
≥7.0	39 (95)
5.0–6.9	2 (5)
Multiple enlarged LLNs on one side^a^	11 (28)
Bilateral enlarged LLNs^a^	3 (8)
Restaging MRI	Whole cohort (*n* = 41) *n* (%)
Mean short-axis size of largest LLN (mm)	8 ± 3
Largest LLN (mm)	
≥7.0	22 (54)
4.1–6.9	16 (39)
≤4.0	3 (7)

MRI, magnetic resonance imaging; IQR, interquartile range; LLN, lateral lymph node

^a^As percentage of patients with an enlarged LLN (≥ 7.0 mm)

### Treatment

After neoadjuvant therapy, nine patients (22%) initially pursued a watch-and-wait approach. Ultimately, 39 patients (95%) proceeded with surgical resection of the primary tumor, whereas 2 patients with a clinical complete response of the primary tumor underwent LLND only. One of these patients later experienced regrowth and subsequently required TME surgery. The surgical procedures included abdominoperineal excision (APE) in 29 cases (70%) and a beyond-TME approach in 11 cases (28%). Approximately half of the surgeries (*n* = 22, 54%) were performed laparoscopically, with the remaining half (*n* = 19, 46%) performed using robotic-assisted laparoscopy. Unilateral LLND was performed for 37 patients (90%), whereas 4 patients (10%) required bilateral LLND due to the presence of bilateral LLNs. Of these four patients, three had bilateral enlarged LLNs and 1 had an enlarged LLN on one side and intermediate LLN with malignant features on the other side.

The majority of the patients underwent nerve-sparing LLND (85%), whereas an en bloc resection of the tumor together with the LLN (not nerve-sparing) was performed in six cases (15%). Intraoperative complications occurred in 12 cases (29%), including significant blood loss exceeding 500 ml in four patients (10%). Among these patients, intraoperative bleeding accounted for total blood loss volumes of 1400 mm, 700 mm, and 600 ml in each of the two remaining cases. Injury to the ureter occurred in two cases (5%), and in one patient, the obturator nerve was injured. In no case was laparoscopic surgery converted to open surgery. The median blood loss was 250 ml (IQR, 100–400 ml), with three cases of major blood loss reported (500 ml in 2 cases and 1400 ml in 1 case). The mean surgical time was 330 min for combined TME and LLND, but could not be specified for LLND alone. None of the patients received adjuvant chemotherapy according to the Dutch colorectal cancer guideline.

### Pathology

The pathologic characteristics of the primary tumors and LLNs are described in Table 3. In 15% of the cases (*n* = 6), TME was performed with involved margins (R1). A average of five LLNs were harvested per unilateral resection specimen. In 41% (18/45) of the LLND resection specimens, malignancy was detected in one or more of the LLNs. For one patient, pathologic assessment of the LLND specimen was not reported separately from that of the TME specimen. In the patients with pathologically positive LLNs, the mean short-axis size at restaging was significantly higher than in those without positive LLNs (9.2 vs. 6.9 mm, respectively; *p* = 0.04), whereas the mean short-axis size on primary MRI did not differ significantly between the two groups (12.5 vs. 10.8 mm, respectively; *p* = 0.19). None of those with lateral lymph nodes smaller than 4 mm (*n* = 2) on restaging MRI had a positive lateral lymph node. Of those with a lateral lymph node 4–5 mm in size on restaging MRI (*n* = 6), two were pathologically positive for malignancy (“Appendix 2”).

**Table 3:** Pathology

	Whole cohort (*n* = 41) *n* (%)
ypT Stage^a^	
ypT0	4 (10)
ypT1	1 (3)
ypT2	9 (23)
ypT3	20 (51)
ypT4	5 (13)
ypN Stage^a^	
ypN0	12 (31)
ypN1	22 (56)
ypN2	5 (13)
Resection with clear margins (R0)^a^	35 (85)
Type of tumor	
Adenocarcinoma	38 (93)
Mucinous carcinoma	2 (5)
Signet ring cell	1 (2)
Differentiation	
Well/moderate	31 (95)
Poorly	2 (5)
Not reported	4
Tumor regression grade^a^	
Complete regression	4 (11)
Partial regression	33 (89)
No signs of regression	1 (3)
Not reported	1
Tumor deposits (≥1)	11 (27)
Presence of EMVI^a^	7 (18)
Not reported	2
Presence of lymphatic invasion^a^	8 (21)
Not reported	2
Mean no. of harvested LLNs^b^	5 ± 3
Not reported	4
Positive LLN^b^	18 (41)
Not reported	1

ypT Stage, pathologic T/N-stage after neoadjuvant treatment; EMVI, extramural venous invasion; LLN, lateral lymph node

^a^As percentage of patients who underwent TME surgery (*n* = 39)

^b^As percentage of each unilateral LLND (in which a bilateral LLND accounts for 2 LLNDs) (*n* = 45)

### Post-Procedural Morbidity and Functional Outcomes

Short-term postoperative complications within 30 days were observed in 22 patients (54%; Table 4), 9 of whom had a complication classified as CDC grade 3 or higher. Pelvic abscess (*n* = 8) and superficial wound infections (*n* = 8) were the most common complications. During this period, urogenital complications occurred for three patients, including ureter leakage in two patients. Beyond the 30 days, a ureteral injury was identified in a third patient. Ureteral reconstruction was performed in all three patients. Two of these three patients had undergone a beyond TME procedure, including a total pelvic exenteration in the one patient and the seminal vesicle in the other patient. The remaining patient underwent a bilateral LLND. Three other patients were readmitted due to urosepsis or hydronephrosis.

**Table 4:** Post-procedural morbidity

	Whole cohort (*n* = 41) *n* (%)
Median time to discharge after surgery: days (IQR)	6 (4–12)
Short-term postoperative complications (<30 days)	22 (54)
Pelvic abscess	8
Ileus/gastroparesis	6
Superficial wound infection	8
Urogenital complications	3
Short-term postoperative complications (<30 days) according to CDC grade	22 (54)
Grade 1	4
Grade 2	9
Grade 3a	1
Grade 3b	8
Planned readmittance (until end of follow-up)^a^	13 (32)
Revision stoma	3
Treatment metastases or recurrence	5
Correction herniation (parastomal/perianal/incisional)	3
Ureteral injury repair	3
Unplanned readmission (until end of follow-up)^a^	18 (44)
Pelvic abscess	9
Ileus/gastroparesis	7
Wound complications	2
Urosepsis	3
Urinary catheter at discharge	
No	27 (66)
Yes, however, also before surgery	3 (7)
Yes, new urinary catheter	11 (27)
Urinary catheter (Foley or intermittent) at last follow-up	
No	32 (78)
Yes (permanent or intermittent)	9 (22)
New urinary complaints (of those without catheter)	8 (35)
Problems with emptying the bladder	3 (38)
Frequent urination	2 (35)
Difficulty delaying urination	1 (13)
No urge for urination	1 (13)
Problems with emptying the bladder, frequent urination, and difficulty delaying urination	1 (13)
Not reported	9
Sexual dysfunction (male)	
No sexual dysfunction	1 (13)
Not sexually active	3 (38)
Erectile dysfunction	2 (25)
Ejaculation dysfunction	1 (13)
Erectile and ejaculation dysfunction	1 (13)
Not reported	16
Sexual dysfunction (female)	
No sexual dysfunction	3 (100)
Not reported	14
LLND-specific complications	
Lymphocele on MRI postoperatively	3 (7)
Lymphedema leg	1 (2)
Function loss upper leg adductors	3 (7)

IQR, interquartile range

^a^Numbers do not add up due to overlap between categories.

At the end of the follow-up period, nine patients (22%) had a newly placed urinary catheter (either self-catheterization or permanent Foley), and among those without a urinary catheter (*n* = 28), eight experienced new urinary complaints, as described in Table 4. Sexual dysfunction was not reported in most cases, although new sexual dysfunction was explicitly mentioned by four patients. Specific to LLND complications, three patients experienced a lymphocele detectable on postoperative MRI or computed tomography (CT). One patient experienced minor leg lymphedema, and three patients had functional impairment of the upper leg adductors, which had not recovered at the last follow-up visit.

A permanent urinary catheter was present more often after beyond-TME surgery (36% vs. 18%; *p* = 0.22) or en-bloc resection of the lateral compartment (33% vs. 20%; *p* = 0.47), but the difference did not reach statistical significance. Bilateral dissection resulted in a permanent urinary catheter rate comparable with that for unilateral dissection (25% vs. 22%; *p* = 0.88).

### Oncologic Outcomes

**Fig. 2:**
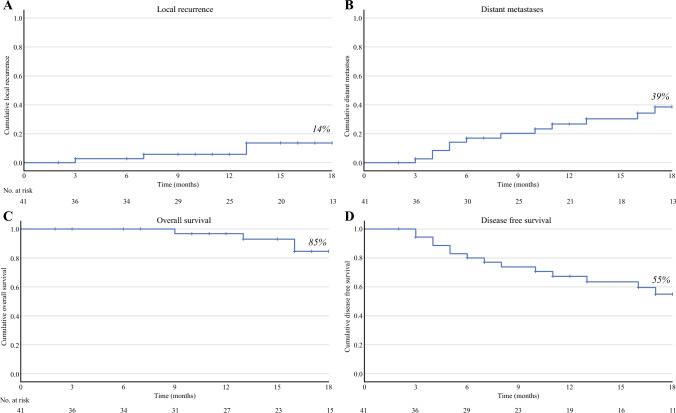
Survival outcomes. **A** The 18-month local recurrence rate is 14%. **B** The 18-month distant metastases rate is 39%. **C** The 18-month overall survival rate is 85%. **D** The 18-month disease-free survival rate is 55%.

After 18 months of follow-up evaluation, six patients (14%) had experienced an local recurrence (LR; Fig. 2A). Among the six observed recurrences, two were located anteriorly, and four were located presacrally. None of these recurrences occurred in the lateral compartment, resulting in a 0% LLR rate at 18 months. Notably, two of these patients had resections with positive margins (R1), and one had a signet ring cell carcinoma (“Appendix 3”). Distant metastasis developed in 39% of the patients by 18 months (Fig. 2B). Detailed tumor and treatment characteristics of the patients with local and distant recurrences are provided in “Appendix 3”. The 18-month DFS rate was 55% (Fig. 2C), and the OS rate was 85% (Fig. 2D).

## Discussion

This study is the first study to assess intraoperative and short-term postoperative outcomes of minimally invasive LLND post-neoadjuvant irradiation in a Western cohort after standardized training and proctoring. During 2 years, only 41 patients underwent LLND surgery for this low-incidence rectal cancer entity, underscoring the need to assess outcomes. Complication rates and functional outcomes were within acceptable limits for locally advanced distal tumors requiring complex procedures, with few CDC grade ≥3 or LLND-associated complications.

One of the primary concerns among Western surgeons regarding LLND is the risk of significant blood loss due to the need for dissection along the internal iliac vessels and their multiple branches. In the current cohort, the median blood loss was 250 ml, with bleeding more than 500 ml reported in only 10% of the cases. This is comparable with TME-only data such as that from the COLOR-II trial and the MRC CLASSIC trial (median blood loss, 200 ml [7%], significant bleeding).^24,25^

The average surgery (TME+LLND) time was 330 min, which is slightly longer than for TME alone (240–250 min),^24,26^ but considerably shorter than the previously described 531 min for TME and LLND.^23^ These findings suggest that LLND adds approximately 90 min of surgery time without significantly increased bleeding risk and should be interpreted in light of the majority of procedures beyond TME and/or APE.

High APE rates are associated with increased morbidity due to the larger pelvic floor defects from more extensive distal dissection.^27^ Previous studies describe perineal wound problems in up to 40% of APE cases, with major morbidity rates of 10–30%.^28,29^ In the current cohort, the unplanned readmission rate was similarly high (42%), primarily involving wound infections, abscesses, and ileus. The LLND-specific complications included intraoperative damage to the obturator nerve in one patient and motoric dysfunction of the upper leg adductors at end of follow-up period in three patients.

Urogenital complications (e.g., neurogenic bladder) were described in 22% of the cases, which aligns with previous studies with complication rates ranging from 0 to 22%.^23,30,31^ This seems acceptable given the differences in therapy (neoadjuvant [C]RT), population (Dutch vs Asian), high rate of en-bloc resections (15%), and/or bilateral dissections (10%) compared with the previous studies. Nevertheless, three cases of ureteral injury necessitating ureter reconstruction were described, but all these cases were managed by extensive primary surgery, including a beyond-TME procedure in two cases and a bilateral LLND in the remaining case.

Although not standard practice for LLND in the Netherlands, ureteric catheter insertion may be considered when a challenging dissection along the ureter is anticipated. Sexual dysfunction was reported in four cases, but limited documentation hampered further analysis.

Specific elements or phases during the dissection with potential to cause urogenital dysfunction were identified, either intraoperatively by the proctor or during the video meetings, and these were fed back to surgeons to reduce complication rates in the future. The ongoing LaNoReC study will provide more insights on urogenital and sexual dysfunction after LLND, whereas extensive (open) procedures with en-bloc lateral compartment resection will be evaluated separately in a registration cohort.

To minimize surgical complications and improve exposure, the procedure was centralized, and procedures were performed in dual-surgeon teams. Previous studies found a reduced operating time after 21 procedures,^32^ with a 20% increased lymph node yield after 30 procedures.^33^ The LLND learning curve had three phases: the learning phase involving the first 51 cases, the proficiency phase involving 52–83 cases, and the mastery period involving 84 or more case.^34^

Despite efforts to centralize this procedure, each surgeon in the current cohort performed only 2–20 procedures, suggesting that they are still in the early learning phase and highlighting the need for additional proctoring and video review, with the expectation of further improvement in lymph node harvest and complication rates.

The primary goal of adding LLND for patients with enlarged LLNs is to reduce the LLR rate. Although the sample size and follow-up time for this cohort were limited, the 0% LLR rate at 18 months was promising. This appears lower than in previous studies, with Kaplan-Meier curves from Sluckin et al.^2^ and Ogura et al.^3^ suggesting an approximate 10% LLR rate at 18 months after (C)RT and TME. The high pathologic positivity rate of 41% after (C)RT compared with 17–29% after node-picking or partial regional lymph node dissection,^35^ reflects both the advanced nature of this cohort (mean LLN size, 11 mm) and the necessity to perform a complete LLND according to anatomic landmarks. Moreover, the persistence of LLNs on restaging MRI highlights the necessity of performing an LLND, even with the risk of urinary dysfunction given the inferior treatment if an LLR develops. The mean retrieval of five LLNs indicates room for improvement in surgical techniques or pathologic assessment because more than 10 LLNs per unilateral minimally invasive LLND were reported previously.^36^ This issue was addressed during the video meetings, and a protocol was developed for clear communication and optimal harvest of LLNs from a resection specimen by pathologists for future improvement in the LaNoReC. Despite this low LLR rate, the 18-month DM rate of 39% is notably high compared with the rate in previous studies investigating locally advanced tumors, even in light of the 7% rate with synchronous metastases.^37,38^

The RAPIDO study included patients with similar clinical characteristics in terms of cT-stage (cT4: 32% vs. 27%), cN-stage (cN2: 69% vs. 49%), mesorectal fascia involvement (67% vs. 65%), and mrEMVI positivity (36% vs. 32%), but reported a lower DM rate after adding systemic therapy to short-term RT.^37^ Tumor location might have contributed to the higher DM rate^39^ because the tumors in this cohort were located very distally (median of 6 mm from the ARJ), in contrast to those in the RAPIDO study (32% located ≥10 cm from the anal verge).^37^ These findings, combined with the small sample of the current study, raise the possibility that only the most advanced cases (with large LLNs and poor oncologic features) are referred to expert centers for LLND surgery, whereas less extensive tumors may be treated without it.

There is a growing shift toward organ-preserving treatment strategies, with increasing use of total neoadjuvant therapy for locally advanced tumors. In this evolving treatment landscape, restaging and follow-up MRI scans have gained importance, especially for cases with a complete response of the primary tumor. Although the Organ Preservation for Rectal Adenocarcinoma (OPRA) study reported a low incidence of LLNs and recurrence rates,^40^ these findings were based on all stages II and III rectal cancer patients including more proximal tumors, and conclusions about restaging or disappearance were drawn from the entire group with visible LLNs rather than from those with enlarged LLNs. Additionally, the OPRA study was conducted in an era wherein the implications of LLNs were less well-known, which may have influenced adequate recognition and assessment. The MRI scans were not re-reviewed for this report, which inevitably has affected its reliability. In our cohort, downsizing of LLNs to 4 mm or smaller, as suggested by Ogura et al.,^4^ occurred in only three cases, but downsizing to 5 mm or smaller carried a significant risk of pathologic nodal positivity. This underscores the critical role of primary MRI for LLND selection and emphasizes the need for caution in omitting LLND in cases of reduced LLN size.

This study had several limitations. First, the small cohort size, reflecting the rarity of the disease, raises concerns about whether radiologists consistently recognize enlarged LLNs because previously only 41% were identified.^41^ Even when enlarged LLNs are detected, not all patients may be referred to expert centers for LLND surgery.

Additionally, the descriptive nature of this study and its specific patient characteristics limit comparison with previous studies on distally located, locally advanced rectal cancer. The newly implemented LLND technique and retrospective data collection hamper the assessment of outcomes not routinely reported during follow-up evaluation, such as sexual or urinary complaints.

Despite its limitations, this study is the first to report these outcomes in a Western population during an early learning period after extensive surgical training. The participation of nearly all LLND surgeons provides a comprehensive overview of current practice and offers valuable insights for preoperative counseling. The currently recruiting LaNoReC trial will provide more detailed data on urogenital and long-term oncologic outcomes.

## Conclusion

Minimally invasive nerve-sparing LLND by trained surgeons shows acceptable complications rates, making it a safe procedure. No LLRs occurred, and the pathologic positivity rate of LLNs in the resection specimen (41%) underscores the potential oncologic value, particularly for locoregional control.

## Figures and Tables

**Fig. 3:**
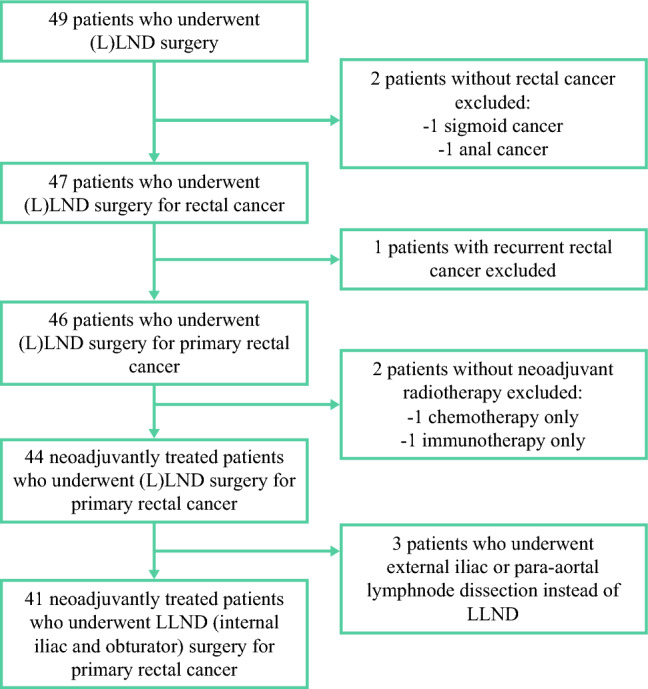
Flowchart of patient selection

## Appendix 1

See Fig. 3.

## Appendix 2

See Table 5.

**Table 5:** Explorative analysis of nodal positivity based on short-axis diameter on primary or restaging MRI

Short-axis size (mm)	Primary MRI *n* (%)	Positive LLNs (pathology) *n* (%)	Restaging MRI *n* (%)	Positive LLNs (pathology) *n* (%)
<4 ≥4	0 41 (100)	0 17 (41)	2 (5) 39 (95)	0 (0) 17 (44)
<5 ≥5	0 41 (100)	0 17 (41)	8 (20) 33 (80)	2 (25) 15 (45)
<6 ≥6	1 (2) 40 (98)	0 (0) 17 (43)	15 (37) 26 (63)	2 (13) 15 (58)
<7 ≥7	2 (5) 39 (95)	0 (0) 17 (44)	19 (46) 22 (54)	4 (21) 13 (57)
<8 ≥8	6 (15) 35 (85)	2 (33) 15 (43)	24 (59) 17 (41)	8 (33) 9 (53)
<9 ≥9	13 (32) 28 (68)	3 (23) 14 (50)	26 (63) 15 (37)	9 (35) 8 (53)
<10 ≥10	17 (41) 24 (59)	5 (29) 12 (50)	29 (71) 12 (29)	11 (38) 6 (50)

LLNs, lateral lymph nodes; MRI, magnetic resonance imaging

## Appendix 3

See Table 6.

**Table 6:** Characteristics of patients with recurrence at end of follow-up period

LR	DM	cTNM stage	NA treatment	W&W	Uni- or bilateral LLND	Beyond TME	Tumor perforation	R1 resection	Type of tumor	pTNM stage
1	1	cT3cN2M0	CRT + boost	N	Uni	–			Adeno	pT3N1bMx
2	2	cT3dN2M0	CRT	N	Uni	Seminal vesicle	Yes	Yes	Adeno	pT4aN1aMx
3	3	cT4bN1cM0	CRT	N	Uni	Back exenteration			Adeno	pT3N1bMx
4	4	cT3bN2M0	CRT	N	Uni	–			Signet cell	pT3N2bMx
5		cT3aN2M0	Induction + CRT	N	Uni	–			Adeno	pT2N0Mx
6		cT4bN2aM0	RAPIDO	N	Uni	Partial prostate and seminal vesicle		Yes	Adeno	pT4bN2aMx
	5	cT3N2M0	CRT	Y	Bi	–			Adeno	pT2N2aMx
	6	cT4bN1cM1	Induction + CRT	N	Uni	–			Adeno	pT4bN1aM1a
	7	cT3cN2M0	CRT	N	Bi	–			Adeno	pT3N1aMx
	8	cT3N1bM0	CRT	N	Uni	–			Mucinous	pT0N0Mx
	9	cT4bN2Mx	CRT	N	Uni	–	Yes	Yes	Adeno	pT3N1Mx
	10	cT4bN2M0	CRT	N	Bi	–			Mucinous	pT4bN1cMx
	11	cT3cN2aM0	CRT	N	Uni	–			Adeno	pT3N2Mx
	12	cT3bN2M0	Induction + CRT	Y	Uni	–			Adeno	pT3N1Mx
	13	cT2N0M0	CRT	N	Uni	NA (LLND only)			Adeno	pT2NxMx

LR, local recurrence; DM, distant metastases; cTNM stage, clinical tumor nodal and metastatic stage; NA treatment, neoadjuvant treatment; W&W, watch and wait; LLND, lateral lymph node dissection; TME, total mesorectal excision; R1, involved resection margins; pTNM stage, pathologic tumor nodal and metastatic stage; CRT, chemoradiation therapy; Adeno, adenocarcinoma; RAPIDO, randomized multicentre phase 3 study of short-course radiation therapy followed by prolonged preoperative chemotherapy and surgery
